# Effect of the App-Based Video Guidance on Prenatal Pelvic Floor Muscle Training Combined with Global Postural Re-education for Stress Urinary Incontinence Prevention: A Protocol for a Multicenter, Randomized Controlled Trial

**DOI:** 10.3390/ijerph182412929

**Published:** 2021-12-08

**Authors:** Lei Gao, Di Zhang, Shiyan Wang, Yuanyuan Jia, Haibo Wang, Xiuli Sun, Jianliu Wang

**Affiliations:** 1 Department of Obstetrics and Gynecology, Peking University People’s Hospital, No. 11, Xi-Zhi-Men South Street, Xi Cheng District, Beijing 100044, China; 1911110389@bjmu.edu.cn (L.G.); 1911110388@bjmu.edu.cn (D.Z.); 0062043910@bjmu.edu.cn (S.W.); jiayuan@bjmu.edu.cn (Y.J.); wangjianliu@pkuph.edu.cn (J.W.); 2 The Key Laboratory of Female Pelvic Floor Disorders, Beijing 100044, China; 3 Clinical Research Institute, Peking University, Beijing 100191, China; wanghb_pucri@bjmu.edu.cn

**Keywords:** pelvic floor muscle training, global postural re-education, pregnancy, stress urinary incontinence

## Abstract

Background: As the effectiveness on stress urinary incontinence (SUI) prevention of pelvic floor muscle training (PFMT) for pregnant women has been inconclusive, we are planning to conduct a trial to evaluate a video program designed for prevention of SUI developed through combining PFMT with global postural reeducation (GPR). Methods: As a randomized controlled trial, eligible participants will be randomized (1:1) into an exercise group and a control group to perform PFMT regularly following video guidance or with no intervention, respectively. The experimental stage will be from the 16th gestation week (GW) to the 12th month postpartum, with eight appointments at the 16th, 28th, 37th GW, delivery, the 6th week and the 3rd, 6th, and 12th month postpartum. Data will be collected regarding urinary leakage symptoms, the stress test, the modified Oxford Scale, pelvic floor ultrasound, perineal laceration classification at delivery, neonatal Apgar score, and questionnaires (PISQ-12, ICIQ-UI SF, I-QOL, OABSS). The primary outcome is the occurrence of the symptomatic SUI and positive stress test at the 6th week postpartum. Discussion: This protocol is anticipated to evaluate the efficacy of the intervention via video app for the design of a future randomized control trial (RCT). Trial registration: The trial has been registered at Chinese Clinical Trial Registry (registration number: ChiCTR2000029618).

## 1. Introduction

Stress urinary incontinence (SUI) is defined as “involuntary leakage of urine when abdominal pressure increases” [1]. The prevalence of SUI at the 8th week postpartum was reported to be 25.7% [2], which exerts a widespread negative impact on women’s health. Pregnancy and delivery have been proven to play important roles for SUI development because it weakens pelvic floor muscle (PFM) strength [3]. Although reducing delivery injuries is the key to prevent SUI, there is currently no consensus among medical society on the effective method for SUI prevention during pregnancy.

Pelvic floor muscle training (PFMT) has been recommended as the first optional treatment of SUI [4], while the effect of prenatal PFMT on SUI prevention is inconclusive. Reilly et al. [3] conducted a randomized control trial (RCT) with 268 primiparas women, and concluded that women in exercise groups who performed PFMT regularly had lower SUI occurrence at the 3rd month postpartum than the control group. However, Boyle et al. [5] suggested that PFMT during pregnancy did not prevent urinary incontinence according to a systematic literature review and meta-analysis of RCTs. Some authors pointed out that the diversity of different studies on the compliance of the patients to PFMT and the procedural correctness may accounted for the inconsistent results [6,7,8,9,10].There currently is no uniform protocol for PFMT during pregnancy, and the protocols vary widely across publications [6,7,11]. How to have the patients perform PFMT voluntarily with high compliance is still a challenge, and the optimal number of contraction repetitions, the time and duration of PFMT contraction, and the frequency of doing PFMT are the determinants of a PFMT protocol to its effectiveness.

Global postural reeducation (GPR) was projected to correct postural misalignments by stretching the muscle chains. As the spine is a continuous boney structure that is closely associated with abdominal and pelvic dynamics, it is commonly recognized that spine restructure through posture reeducation would benefit the functions of the PFMs. Our previous study demonstrated that the spinopelvic skeletal shape had a close relationship with pelvic floor dysfunction [12]. In 1981, Souchard first developed GPR in France, aiming to keep the normal posture to recover muscular strength. Fozzatti et al. [13,14] recruited SUI patients for treatment by the GPR and PFMT, and demonstrated that both GPR and PFMT were effective alternatives for the treatment of SUI, while GPR was significantly more effective. 

To explore a more effective exercise regimen for SUI prevention, we developed a training program through combining PFMT with GPR training (PGT program) under the collaborative efforts from several urogynecologists and sports experts from Peking University.

Patient’s compliance is another important factor for the effectiveness of PFMT. Face-to-face training is time-consuming and needs frequent visits to relevant facilities, which therefore further decreases the adherence to the training program. In addition, it is quite hard to organize face-to-face training programs during the COVID-19 pandemic. For a long time, what has been bothering the urogynecologists is how to achieve intensive supervision and encourage participants to keep high compliance. Popularization of mobile phones makes almost everyone accessible via mobile devices, which enabled women to connect to the world from at home; thus, a PGT video guidance on mobile apps can create potentiality to enhance the adherence and compliance of the patients who can perform PFMT and GPR at home.

Therefore, an app-based video program was developed with the collaboration of software engineers. The video program tends to guide the patients to do regular PFMT and GPR at home or any private room. To make the patients’ compliance evaluable during the COVID-19 pandemic, we integrated a high-intensive online supervision module into the video program. We conduct this multicenter RCT to evaluate the effectiveness and safety of the video program on the prevention of postpartum SUI. 

## 2. Materials and Methods

### 2.1. Study Overview

The study is designed as a two-arm, parallel, RCT designed following the Consolidated Standards of Reporting Trials (CONSORT). Participants will be recruited from the obstetrics departments of ten hospitals (the centers) including Peking University People’s Hospital, Peking University International Hospital, Peking University Shenzhen Hospital, Beijing Obstetrics and Gynecology Hospital, Fangshan Maternal and Child Health Hospital of Beijing, Fengtai Maternal and Child Health Hospital of Beijing, Mentougou District Hospital of Beijing, Zhengzhou Central Hospital Affiliated to Zhengzhou University, Luohe Central Hospital of Henan Province and The First Obstetrics Hospital of Shanghai. The ten hospitals are selected as the centers because (1) they have enough patients to recruit the minimum number of patients proposed for each center, and (2) the investigators at those centers are knowledgeable and experienced in the identification and treatment of the SUI. 

### 2.2. Inclusion Criteria

Pregnant women will be eligible for recruitment if they are: (1) 20 to 40 years of age, (2) primipara with a singleton pregnancy of less than 16th gestation week (GW), and (3) willing to participate in the study and understand the research procedure well. 

### 2.3. Exclusion Criteria

**Figure 1:**
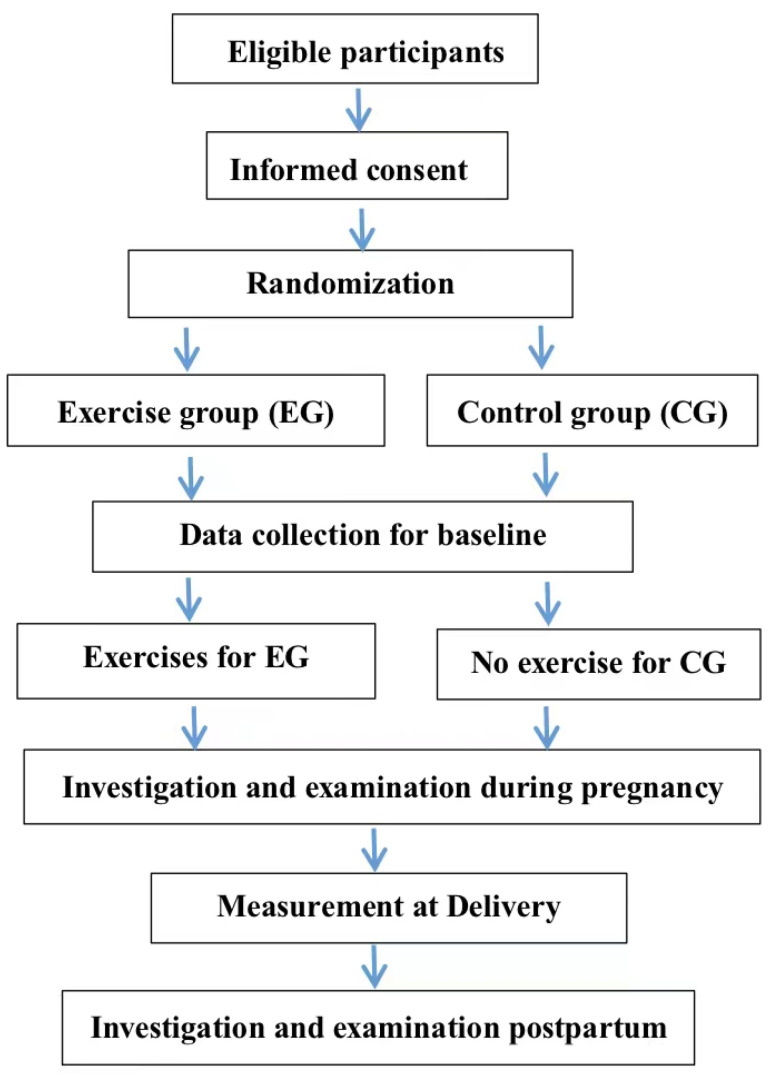
Study flow.

In the first enrollment examination, participants will be excluded if they were confirmed: (1) having serious complications (including uncontrolled brain, lung, heart, liver, kidney, and mental illness; coagulation dysfunction; or obvious cognitive dysfunction) that may be aggravated by the intervention, (2) having SUI or pelvic floor organ prolapse (POP) according to pelvic organ prolapse quantitation (POP-Q) staging, (3) having history of cervical insufficiency, recurrent miscarriage, and induced labor, (4) unable to finish all follow-ups, (5) infecting COVID-19, and/or (6) unable to provide consent in writing (Figure 1). Each participant will be recorded as “drop off from trial” if she suffers from miscarriage, serious complications, or missed all follow-ups. Cesarean will not be the endpoint of the experimental follow-ups.

### 2.4. Randomization and Allocation Concealment

A randomization list will be created by a statistician from Peking University for each center before the start of the research using SAS 9.4 (SAS Institute, Cary, NC, USA). The numbers of participants in EG and CG are balanced, with a 1:1 ratio, and put into blocks of four. A unique study ID will be given to each participant. 

The randomization result for EG and CG are sealed in the ID-matching envelopes. The grouping details will be concealed to the investigators who will account for group allocation, recruitment, informed consent, and data collection. Eligible women are informed of grouping allocation via opening the sealed envelope after signing the informed consent. It is not feasible to blind to participants, urogynecologists and physiotherapists engaging in the intervention. However, all the investigators to perform patient evaluation and data analysis will be masked.

### 2.5. Interventions

All participants in EG and CG will have same routine obstetric prenatal care following the standard clinical procedures. No intervention will be given to the participants in CG. Participants in EG will perform PGT from the 28th GW to their delivery following the video guidance on mobile app. They will be instructed to download and install the app by research assistants.

The app provides health education and a PGT video. Watching the health education video, participants can learn the pelvic floor anatomy and function and are informed about the negative impact of pregnancy and delivery on PFM. The PGT video includes PFMT and GPR programs designed collaboratively by the urogynecologists and the sports experts. The GPR combines global posture and respiratory training with PFMT to enable participants to exercise PFMT while posturing.

PFMT mainly focuses on voluntary contraction and relaxation of PFM around urethra, vagina and rectum. It provides instruction on coordinating PFM contraction and relaxation by exhalation and inhalation of a breathing circle, respectively, while avoiding the involvement of abdominal muscles. Participants need to contract the PFM first with moderate strength and upholding for 6–8 s, followed by 6–8 s PFM relaxation (the primary PFMT procedure), then repeat primary procedure five times to train the type I muscle fibers. As the second step, participants are asked to do quick maximum PFM contraction and relaxation in 1 s for five times, followed by 10 s of relaxation (the secondary PFMT procedure). The secondary PFMT procedure will be repeated for eight times to train the type II muscle fibers. Both the primary and secondary PFMT procedures need to be repeated for three sets in an exercise circle. Participants in EG need to perform PFMT twice a day following the instruction of the video program. 

The GPR requires the participants to perform respiratory training, PFMT, and global posture simultaneously. It consists of four sets of exercises, each lasting about 30 min and composed of four groups of postures. The postural intensity increases from the first set to the last one. The physiotherapists will instruct the participant to do the proper set of exercises at a specific period according to her gestation weeks. Respiration training during the whole exercise is essential to expand the chest and abdominal cavity that aims to strengthen the relevant muscles. Participants will be guided to exhale deeply while contracting muscles and inhale deeply while relaxing muscles. The program starts with respiratory training in the supine position while performing one set of PFMT, followed by 20 min of aerobic exercise by keeping global postures such as bridge pose, cat pose, child’s pose, and mountain pose, and in-between one set of PFMT is performed. The exercise of GPR will be ended with meditation in the supine position and one set of PFMT will be performed simultaneously. 

The twice-a-week GPR was originally programmed to be performed once following guidance of the app at home and once under face-to-face guidance of the physiotherapists. In consideration that it is not safe to organize face-to-face guidance in training rooms during COVID-19 pandemic and the need to regulate the postures and procedures and collect data to evaluate the compliance of the participants, we modified the protocol to organize video-trainings for EG participants to perform GPR under the living guidance of the physiotherapists on-video via Zoom meetings once a week. Four Zoom meetings will be scheduled for video training in each week, each focusing on one of the four exercise sets. An individual participant will be invited to visit one of the Zoom meeting to practice the exercises appropriate to her GW. Each video-meeting can be divided into several classes according to the number of participants, with each limited to 10 persons, so as to facilitate the physiotherapists to give enough care to each participant and to maximize the effectiveness. The site assistants will notify each participant the Zoom link specially for her in each week in advance, but keep her blind of the other Zoom meeting details to avoid protocol bias. The site assistant will also record the attendance of each participant in terms of the attendance time, the procedural and intensity compliance, and the adverse events, etc. Any participant absent at the scheduled class will be reminded to join the next class on the same exercise set so that she can “catch up” the scheduled one in the following Zoom meeting. The scheduled class will not be cancelled even with only one participant. The role of the physiotherapist is to guide participants in the class to accomplish the full exercise set and to regulate any inappropriate acts. To protect participants’ privacy, no video and audio records are taken and preserved during video-conference, which are fully informed to and consented by the participants.

Any pregnant participant experiencing abnormal symptoms (e.g., abdominal pain, vaginal bleeding, abnormal fetal movement, etc.) will contact the site assistant in each hospital, and the assistant will refer them to the obstetric department as soon as possible, followed by an assessment of whether they should continue the intervention or not. An anticipated adverse event report will be prepared and sent to the principal investigator (PI). 

### 2.6. Data Collection and Management

Data regarding the clinical examination during the follow-ups will be collected from all participants in both groups. A questionnaire of demographic characteristics including the age, body mass index (BMI), educational status, working type, working posture, toilet type, PFMT history, constipation history, smoking history, number of gestations, number of abortions, family history of SUI, and family history of POP will be collected from the participants with gestation ≤ 16 GWs. The Prolapse/Urinary Incontinence Sexual Questionnaire short form (PISQ-12) [15] will be filled by all participants. Data regarding the MOS, and genital hiatus (gh) and perineal body (pb) measured by pelvic organ prolapse quantitation (POP-Q) of all the participants will be provided by the urogynecologists. Trans-perineal ultrasound using the proprietary software 4D View v 10 (GE Kretz Medizintechnik) will be conducted for all the participants to examine the residual urine, the thickness of pb, detrusor muscle, and levator ani muscle (LAM), and the diameters of the levator hiatus and levator hiatus area. Urinary leakage symptoms, and frequency of PFMT and GPR performance, will be taken from all the enrolled participants respectively at the 28th and 37th GW and at the delivery. Those who have urinary leakage symptoms will fill out the Incontinence Quality of Life (I-QOL) [16] and the International Consultation on Incontinence Questionnaire-urinary incontinence short form (ICIQ-UI SF) [17], and Overactive Bladder Symptom Scores (OABSS) [18] will be assigned to participants who have symptoms of urgency urinary incontinence (UUI). Evaluation of the PFM strength via MOS, stress test, and gh and pb measured by POP-Q will be conducted on all participant in both groups on the 37th GW. The delivery type, the duration of the second labor stage, the perineal laceration classification, the visual analog scale (VAS) score, neonatal Apgar score and the neonatal weight will be recorded during delivery. The visual analog scale (VAS) will be scored at 24 h and 48 h after the delivery to measure the pain degree by scoring 0 to 10. Fetal/neonatal safety will be evaluated with indicators such as the fetal movement, obstetric ultrasound, fetal heart rate, electronic fetal monitoring, neonatal Apgar score, and obstetric examination. 

Four follow-ups will be conducted on all participants respectively on the 6th week and in the 3rd, 6th and 12th month postpartum. To conduct the multiple physical valuations in a time-saving way, it is proposed to check the participant in the following sequence: first, check the stress test, POP-Q, MOS when the bladder is half-full; second, conduct the pelvic floor ultrasound examination and the pelvic floor electrophysiological test (Electron-IC Concept Lignon Innovation Co., Montpellier, France) with the emptied bladder; and last, fill the PISQ-12. The pelvic floor electrophysiological indicators mainly include the vaginal resting pressure (cmH2O), the vaginal maximum contraction pressure (cmH2O) and PFM fatigue (%). I-QOL, ICIQ-UI SF, and OABSS will be provided to participants who show symptoms of urinary leakage or UUI as appropriate (Table 1).

Gynecologic examination, pelvic floor ultrasound, pelvic electromyography, and fetal safety will be evaluated respectively by the urogynecologists, ultrasound professors, electromyography nurses, and obstetric professors in each hospital, who will be trained in the Peking University People’s Hospital before the launch of the study. A program that is preset into the video will record times for the full-playing of the video and automatically transfer the data into the database as the training frequency for each participant. The site assistants will confirm the training frequency of each participant in EG every week by checking the records from the online-guided software, exercise diary, and the video-training. Accomplishment of PFMT will be identified for each participant should she accomplish more than 80% of the training programs prescribed for every week. 

Data input devices will be provided to the hospitals to be involved in the research. Paper files will be prepared and kept for each participant to record all data to be collected in the full study stage. Exercise diaries from participants in EG will be used to evaluate their PFMT frequency and training intensity. The database will be protected from access by a password, and the final dataset will only be accessible to the PI, and, under authorization of the PI, to the study coordinator. Desensitized data are available for research purpose only per the permission of the PI. 

### 2.7. The Outcomes

The primary outcome of this study is the occurrence of SUI at the 6th week postpartum, which will be diagnosed based on urinary leakage when abdominal pressure increases or positive stress test. A positive result of the stress test is defined as involuntarily urinary leakage during Valsalva or coughing in the lithotomy position.

The secondary outcomes include: (1) the occurrence of SUI at the 12th month postpartum; (2) the PFM strength at the 6th week and 12th month postpartum, which will be measured with the MOS and pelvic floor electrophysiological test.

### 2.8. Sample Size

The sample size was calculated using PASS 2019 software. Qi et al. [2] reported 25.7% of the 8-week postpartum SUI occurrence. Because there is lack of SUI occurrence in 6-week postpartum to refer to, we hypothesize the 6-week postpartum SUI occurrence is about 25.7% and the occurrence of SUI in EG will dropped to 15% after the trial. Based on 10.7% of occurrence difference between two groups at 6-weeks postpartum, a total of 586 participants (293 per group) is required to provide 90% statistic power at the two-sided significance level of 0.05. In considering 20% of the possible dropout, we decided to recruit 734 participants (367 subjects in each group) for the trial.

### 2.9. Statistical Analysis

Statistical analysis will be conducted using SPSS version 23.0 and the assumed significance level was 5%. Categorical variables will be described as numbers and percentages. If continuous variables followed a normal distribution, data will be represented as means ± standard deviations (SDs). Otherwise, medians (P25, P75) will be calculated when continuous variables do not follow a normal distribution. For the primary outcome, we will compare the occurrence of SUI at 6-week postpartum between EG and CG by chi-square test or Fisher’s exact test as appropriate. For secondary outcomes, the counting data will be analyzed by logistic regression and the quantitative data will be compared by linear regression as appropriate.

Statistical analyses will be conducted on an intention-to-treat (ITT) basis by the statistician at Peking University Clinical Research Institute. Data analyses of EG will also include those regarding the participants’ adherence to the protocol with at least 80% participation to be the indicator of task achieved.

The outcome analysis will be conducted with multiple imputations to take missing values into account. The statisticians will perform sensitivity analyses of the outcome first by imputing missing values as failures and then by using complete cases.

## 3. Discussion

Pregnancy and delivery are important risk factors in the development of SUI because it potentially weakens and damages nerves, PFM, and connective tissues [10,19]. The prevalence of SUI was reported to be 18–75% in late gestation and approximately one-third at postpartum, which causes an enormous social and health burden to women [6]. It has been suggested that prenatal SUI is a strong predictor for suffering from long-lasting SUI [20]. Therefore, SUI-targeted prevention management during pregnancy may be an effective way to prevent SUI in the future. Some urogynecologists have explored the protective effect of PFMT during pregnancy on the pelvic floor. However, their results were inconsistent according to differences in protocol, SUI diagnosis criteria and the time-point of follow-up [21]. 

The correctness and compliance of PFMT may be the key factors for the result inconsistencies of those studies [6,7,8,9,10]. Most of the PFMT protocols were based on Kegel exercise, and many studies have just verified the short term effectiveness without clear demonstration on the long-term effectiveness. Based on our practices and in referring to the publications, we believe that the correctness and compliance of PFMT might affect the effectiveness. The optimal number of contraction repetitions, time, and duration of PFMT and the daily training frequency need to be identified. Duchateau et al. [22] proposed that isometric contraction could be used as functional training to inspire stronger strength. When the contraction force reaches 90–100% of the maximum strength, the duration time is better when 6–8 s, followed by the same time of relaxation [23]. In addition, according to 1701 articles, García-Sánchez et al. [24] suggested that: (1) 10–45 min of PFMT per session and 3–7 days per week might inspire the greatest changes of PFM; (2) at least six-week training was needed to reach SUI symptom improvement; and (3) more than 12-week training tended to achieve an obvious decrease in urine loss. The PFMT protocol in our study requires PFMT for 20 min per day and lasts for 12 weeks, matching with what García-Sánchez et al. suggested.

Twenty-seven years after Souchard [13] first proposed GPR in France in 1981, Fozzatti et al. [14] recruited 26 patients with symptoms of SUI to treat with GPR for three months and achieved great improvement of the SUI symptoms at the six-month follow-up. Another two years later, Fozzatti et al. [13] recruited 52 SUI patients and divided them into a PFMT group and a GPR group to perform PFMT and GRP, respectively, for three months, and found that GPR group showed higher adherence to treatment than PFMT group. They concluded that PFMT and GRP were efficient alternatives for treatment of SUI, but the GPR group had higher efficacy. GPR is based on the hypothesis that muscles are organized in chains that contribute to keeping the vertical erect posture [13,25]. GPR aims to correct postural misalignments based on stretching the muscle chains, aiming to keep the normal posture and recover muscular strength. The posture restructuring is expected to benefit the muscle functions. Besides muscles, the spine is also a continuous structure organized in chains that is closely associated with abdominal and pelvic dynamics. Mattox et al. [26] found that the abnormal change in spinal curvature and, specifically, the loss of lumbar lordosis, appeared to be an important risk factor in the development of POP. More thoracic kyphoses were founded in POP women than normal women [27,28], and the change in breathing dynamics and consequent increase pressure in abdomen may account for it. Respiratory regulation is purposed to alter the respiratory dynamics and vary the shape of the abdominal cavity, which helps to adjust the pelvic dynamics and the downward pressure on pelvic organs [29]. In our study protocol, we integrate global posture and respiratory training together as the intervention in the GPR, with which the participants are guided to contract the PFM while exhaling and relax the PFM while inhaling. 

Petros [30] has found that some exercises, such as squatting-based exercises, can produce three directional muscle actions which could pull against pubourethral (PUL) and uterosacral (USL) suspension, thus helping to close urethra. Davenport et al. [6] proposed aerobic exercise during pregnancy could reduce the odds of prenatal and postnatal UI. In referring those thoughts, the urogynecologists and sports experts integrate PFMT, aerobic exercise, and special global postures into the PGT program, which have not been mentioned in papers before.

As we all know, mobile phones are becoming increasingly common, thus the app-based video guidance is easy to achieve with the aid of mobile devices. During the COVID-19 pandemic, adoption of the mobile-device-based PGT video program and Zoom meetings will not only enable the participants to perform PFMT and GPR safely at home, and increase participants’ interests for exercise, but also improve the compliance of participants, because participants can easily perform the 10-min PFMT session in any spare time and at any place. The automatic frequency recording design of the PFMT software is expected to provide better supervision on the compliance. 

This protocol is designed according to referring guidelines, consensus and high-quality studies. As a multicenter RCT, it will involve ten hospitals including tertiary hospitals and maternity hospitals located in several provinces, purposed to achieve more convincing results than other single center protocols [31,32]. 

The possible limits of this trial are that there will be no comparison of the virtual guidance and supervision versus the face-to-face ones because of the COVID-19 pandemic, and that the data may be contaminated due to no effective way to limit the participants in the control group doing PFMT personally. Another limitation of the trial is that the long follow-up time may increase the amount of the missing data.

## 4. Conclusions

In conclusion, this study is expected to help us understand the efficacy and feasibility of the PGT video app and the intervention design for future clinical trials.

## Figures and Tables

**Table 1:** Baseline screening, assessment, and follow-up schedule.

	At Pregnancy				At Postpartum			
	<16th Week	28th Week	37th Week	Delivery	6th Week	3rd Month	6th Month	12th Month
Follow-up schedule (week)	0	± 1	± 1	± 1	± 1	±2	± 2	± 2
Informed consent	•							
Demographic data collection	•							
Urinary leakage symptoms	•	•	•	•	•	•	•	•
Training frequency	•	•	•	•	•	•	•	•
Pb and gh	•		•					
Stress test	•		•		•	•	•	•
POP-Q staging					•	•	•	•
The modified Oxford Scale	•		•		•	•	•	•
The duration of the second labor stage				•				
The perineal laceration classification				•				
VAS				•				
Fetal/natal conditions	•	•	•	•				
Pelvic floor ultrasound	•				•	•	•	•
Pelvic floor electrophysiological test					•	•	•	•
PISQ-12	•				•	•	•	•
ICIQ-UI SF		▲	▲		▲	▲	▲	▲
OABSS		▲	▲		▲	▲	▲	▲
I-QOL		▲	▲		▲	▲	▲	▲

•: Indicates mandatory items; ▲: Investigator will decide whether to perform the test according to clinical signs or clinical evaluation. Pb: perineal body; gh: genital hiatus; POP-Q: pelvic organ prolapse quantitation; VAS: visual analog scale; PISQ-12: Prolapse/Urinary Incontinence Sexual Questionnaire short form; ICIQ-UI SF: the International Consultation on Incontinence Questionnaire-urinary incontinence short form; OABSS: Overactive Bladder Symptom Scores; I-QOL: the Incontinence Quality of Life.

## Data Availability

Not applicable.
